# An Immature *Pachyrhinosaurus perotorum* (Dinosauria: Ceratopsidae) Nasal Reveals Unexpected Complexity of Craniofacial Ontogeny and Integument in *Pachyrhinosaurus*

**DOI:** 10.1371/journal.pone.0065802

**Published:** 2013-06-19

**Authors:** Anthony R. Fiorillo, Ronald S. Tykoski

**Affiliations:** Perot Museum of Nature and Science, Dallas, Texas, United States of America; Raymond M. Alf Museum of Paleontology, United States of America

## Abstract

A new specimen attributable to an immature individual of *Pachyrhinosaurus perotorum* (Dinosauria, Ceratopsidae) from the Kikak-Tegoseak Quarry in northern Alaska preserves a mix of features that provides refinement to the sequence of ontogenetic stages and transformations inferred for the development of the nasal boss in *Pachyrhinosaurus*. The new specimen consists of an incomplete nasal that includes the posterior part of the nasal horn, the dorsal surface between the horn and the left-side contacts for the prefrontal and frontal, and some of the left side of the rostrum posteroventral to the nasal horn. The combination of morphologies in the new specimen suggests either an additional stage of development should be recognized in the ontogeny of the nasal boss of *Pachyrhinosaurus*, or that the ontogenetic pathway of nasal boss development in *P. perotorum* was notably different from that of *P. lakustai*. Additionally, the presence of a distinct basal sulcus and the lateral palisade texture on the nasal horn of the specimen described here indicate that a thick, cornified horn sheath was present well before the formation of a dorsal cornified pad. A separate rugose patch on the nasal well posterior to the nasal horn is evidence for a cornified integumentary structure, most likely a thick cornified pad, on the posterior part of the nasal separate from the nasal horn prior to the onset of nasal boss formation in *P. perotorum*.

## Introduction

The centrosaurine ceratopsid dinosaur *Pachyrhinosaurus* is represented by three species (*P. canadensis* [1]–[3]; *P. lakustai* [4]; *P. perotorum* [5]) known from upper Campanian and lower Maastrichtian deposits of Alberta, Canada and the North Slope of Alaska, U.S.A. All three species are known from material representing multiple individuals, with *P. lakustai* and *P. perotorum* known from bone beds that record mass death assemblages [4], [5]. The most ubiquitous cranial feature shared by mature individuals of all three species is the presence of a massive, thickened, nasal boss that covers most of the dorsal surface of the skull anterior to the orbits. The nasal boss occupies the same portion of the rostrum that bears erect nasal horns or other nasal ornamentation in more basal centrosaurine taxa [6], [7]. Mature specimens of all three species also share the presence of enlarged supraorbital bosses instead of horns, as well as large, laterally curved horns at the P3 locus [8] on the parietal frill. It should be noted that an alternative numbering system was recently proposed for identification of centrosaurine epiparietal processes [9], but that debate is not pertinent to the specimen we describe here.

The three *Pachyrhinosaurus* species are differentiated from one another by combinations of craniofacial and frill ornamentation features, which include relative size and coverage of the nasal boss, presence or absence of a rostral comb on the premaxilla, and presence or absence of a medially directed horn on the P2 locus of the parietal frill [4], [5]. In addition, *P. perotorum* is diagnosed by the presence of a small, anteriorly-directed horn arising from the anterior edge of the transverse parietal bar and overhanging each parietal fenestra of the frill [5]. The species may possibly be diagnosed by a rounded, upturned rostral beak (unique among Ceratopsia), and reduction or closure of the frontal fontanelle [5].

The large sample of *Pachyrhinosaurus lakustai* specimens preserves parts of individuals from many ontogenetic stages, which allowed Currie et al. [4] to verify that the nasal boss in *P. lakustai* is homologous to the nasal horns and other ornamentation present in other centrosaurine taxa. The sample shows that small, immature nasals are unfused and bear a low, blade-like demihorn that may be marked by extensive neurovascular foramina and grooves of variable orientations. The demihorn in immature *P. lakustai* specimens extend from anterior to the border of the narial aperture to nearly the posterior margin of the nasal at the prefrontal and frontal contacts. Larger but still immature specimens show beginnings of the swelling and rapid expansion and build-up of the nasal boss, and only specimens at least two-thirds of ‘adult’ size exhibit a tall, transversely wide boss structure marked laterally with dorsoventral grooves that give the sides of the nasal boss a palisaded texture. It was inferred by Currie et al. [4] that the ontogenetic development of the nasal boss followed a pattern of nearly isometric growth until attaining approximately two-thirds of ‘adult’ size, followed by rapid lateral migration of the paired demihorns accompanied by massive deposition of bone between them. It was further postulated [4] that the posterior medially angled surface of the demihorn present in immature specimens grew upward and further posteriorly to eventually take the form of the transversely wide posterior rim of the boss.

**Figure 1:**
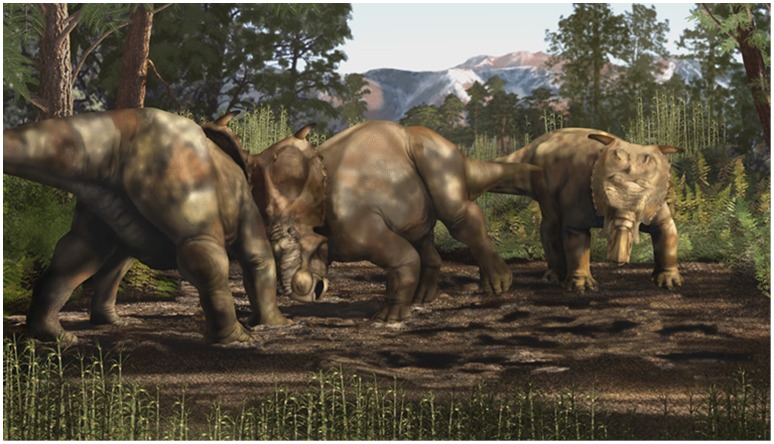
Artistic rendering of *Pachyrhinosaurus perotorum* engaged in head-butting/pushing behavior. In the first description of *Pachryhinosaurus* by Sternberg [1], he speculated that the enlarged nasal boss in the taxon might have been used in head battering or pushing behavior, an idea emphasized by this image of two *Pachyrhinosaurus perotorum* sparring with their craniofacial bosses, while a third looks on. Artwork by K. Carr.

In addition to descriptions of osteology and inferences of ontogeny of the craniofacial bosses in *Pachyrhinosaurus*, these massive and visually bizarre structures have prompted speculation and hypotheses of function, behavior and soft-tissue reconstruction in this and related centrosaurine taxa. It was first suggested by Sternberg [1] that the craniofacial bosses in *Pachyrhinosaurus* were an adaptation for head-butting or head-pushing behavior (Figure 1), and that the bosses were covered in life by cornified skin. More radical reconstructions of *Pachyrhinosaurus* envisioned a large, rhinoceros-like dermal horn arising from the nasal boss, although such a structure was viewed with skepticism by others [4], [10], [11], [12].

**Figure 2:**
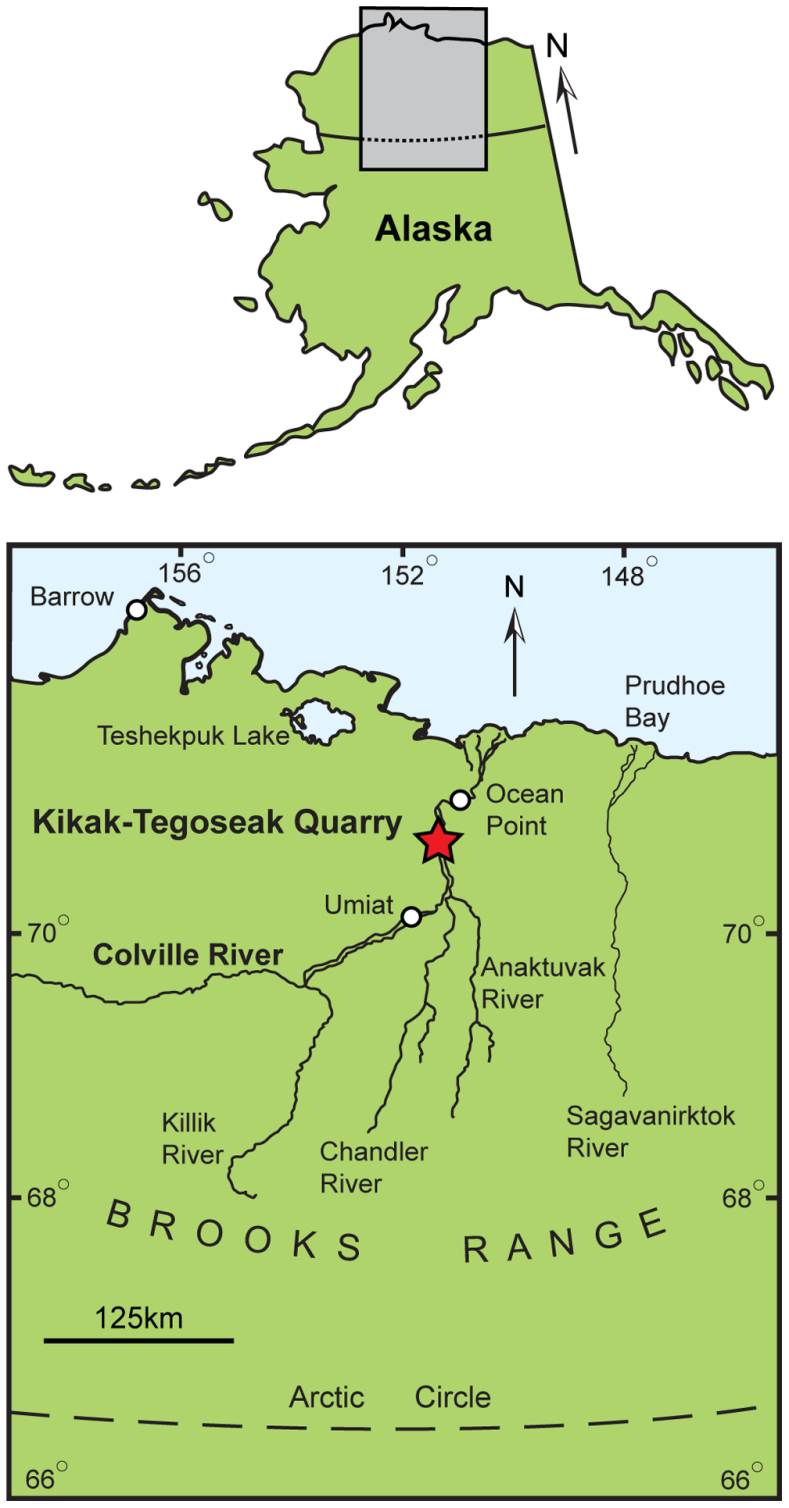
Map showing approximate location of the Kikak-Tegoseak Quarry, North Slope, Alaska, U.S.A. Kikak-Tegoseak Quarry indicated by red star.

Here we describe and discuss the implications of a small, incomplete nasal (DMNH 21460) referred to *Pachyrhinosaurus perotorum* from the Kikak-Tegoseak Quarry of Alaska (Figure 2), the type locality of *P. perotorum* [5]. The Kikak-Tegoseak Quarry is a monodominant bonebed deposit [13] with a minimum of eleven individuals represented in the quarry, based upon the number of occipital condyles currently known from the site (including that in the nearly complete skull DMNH 22558). The specimen was collected as a float block found within a meter of the main Kikak-Tegoseak Quarry edge. We are confident that the new specimen can be referred to *P. perotorum* and that it does not represent a second ceratopsid taxon from the site. The specimen is notable because it comes from a smaller, relatively immature individual, contrary to previous published statements about the individuals from the quarry all being of similar ‘adult’ ontogenetic stage [13] and therefore expands the known age profile of this taxon from the site. The specimen has dorsally enlarged nasal ornamentation marked by lateral grooves and ridges (palisades), but is mediolaterally narrow and anteroposteriorly short. This differs from the ontogenetic trajectory of nasal boss development hypothesized for *P. lakustai* [4]. In addition, the posterior part of the nasal preserves unusual bone texture and structure that provide evidence for a degree of integument complexity not previously recognized in other species of *Pachyrhinosaurus*.

### Institutional Abbreviations

**CMN**, Canadian Museum of Nature, Ottawa, Ontario, Canada; **DMNH**, Perot Museum of Nature and Science, Dallas, Texas, U.S.A, formerly the Museum of Nature and Science; **TMP**, Royal Tyrrell Museum of Paleontology, Drumheller, Alberta, Canada.

### Geological Setting and Locality Information

The Kikak-Tegoseak Quarry is located in the extensive exposures of the Prince Creek Formation along the a bluff overlooking the Colville River, North Slope Borough, Alaska, USA. (Figure 2). The Prince Creek Formation is an alluvial unit comprised of sediments shed northward from the rising Brooks Range from late Campanian to Paleocene time [13]. Radioisotopic dates derived from multiple tuff beds throughout the section of the Prince Creek Formation range from 68 Ma to 71 Ma, with an average estimate of 69.1+/−0.3 Ma [13], [14]. Palynological samples from the Kikak-Tegoseak Quarry itself correlate well with the radioisotopic data, showing an Early Maastrichtian assemblage [13].

## Materials and Methods

The specimen, DMNH (Perot Museum of Nature and Science) 21460 was collected under BLM permit number AA-86367. The fossil was extracted from its surrounding rock using mechanical pneumatic air tools (air-scribes), and Butvar B-76 was used as both a consolidant and an adhesive in stabilizing and reassembling the specimen. The specimen is housed in the collections of the Perot Museum of Nature and Science, in Dallas, Texas, USA.

### Description

**Figure 3:**
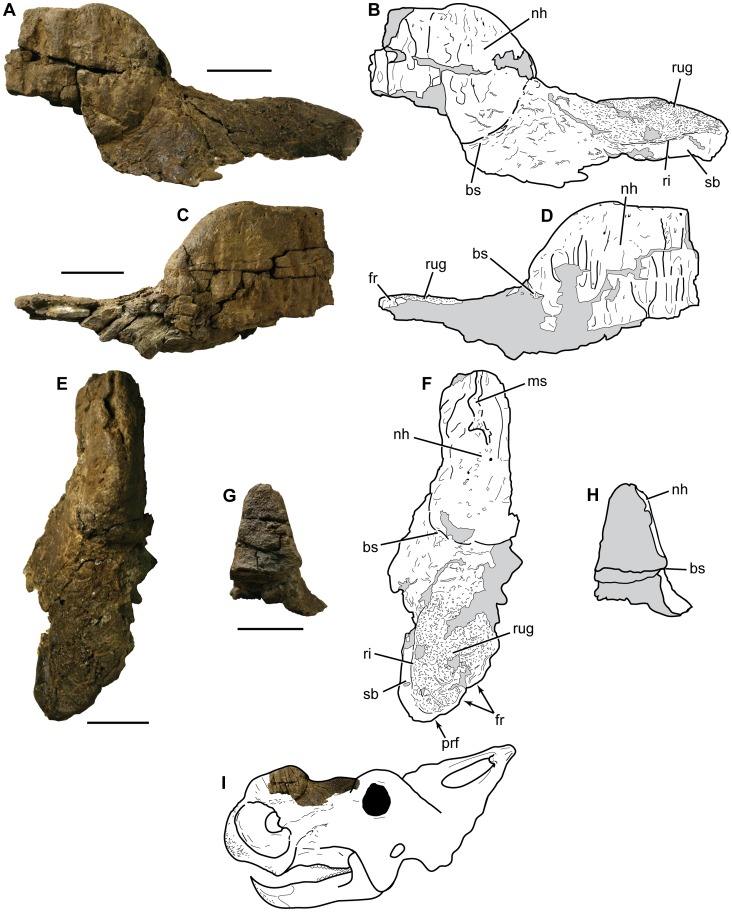
Photographs and interpretive line drawings of DMNH 21460, *Pachyrhinosaurus perotorum* incomplete nasal. A. Nasal in left lateral view. B. Line drawing of nasal in left lateral view. C. Nasal in right lateral view. D. Line drawing of nasal in right lateral view. E. Nasal in dorsal view. F. Line drawing of nasal in dorsal view. G. Nasal in anterior view. H. Line drawing of nasal in anterior view. I. Hypothetical ‘subadult’ *Pachyrhinosaurus perotorum* skull outline with DMNH 21460 superimposed to show approximate location of specimen in the skull. Abbreviations: bs, basal sulcus; fr, frontal contact; ms, median sulcus; nh, nasal horn/incipient nasal boss; prf, prefrontal contact; ri, ridge laterally bounding rugose area; rug, rugose patch on dorsal surface of nasal; sb, smooth bone. Gray fill indicates broken bone surfaces or cracks. Scale bars equal 5 cm.

The incomplete specimen DMNH 21460 consists of parts of both right and left nasals fused together to form a single functional unit, hereafter referred to as a singular nasal (Figure 3). The specimen was broken through the transverse plane anteriorly, the horizontal plane ventrally, and the posterior portion through a nearly sagittal plane just to the right of the midline. It includes the posterior part of the nasal horn, the dorsal surface between the horn and the left-side contacts for the prefrontal and frontal, and some of the left side of the rostrum posteroventral to the nasal horn. The unusual morphology of the specimen led to initial uncertainty as to its position in the skull and whether it also encompassed parts of the frontals, prefrontals, or other supraorbital elements. There are no visible sutural contacts in the specimen, which argues against there being additional elements being incorporated in it. The ventrolaterally directed margin of the specimen’s ventrolateral and posterolateral edges is inconsistent with the raised anterodorsal part of the orbital rim seen in specimens of *P. lakustai* and other centrosaurines [4], [8], [9], [12], [15]. A broad, gently convex area of rugose-textured bone covering the posterior part of the specimen bears no similarity with the raised supraorbital horns of immature centrosaurines [4], [12], [15], [16]. It is also very different from the highly derived supraorbital boss of mature specimens of *Pachyrhinosaurus* [1], [2], [4], [5]. The nearly vertical, faintly pitted faces of the posterior bone margins are more consistent with the position and likely position for an abutting contact with the frontals or prefrontals. We therefore determined that the specimen represented only an incomplete nasal, preserving the posterior part of the nasal horn core and the span between it and the frontal and prefrontal contacts.

**Figure 4:**
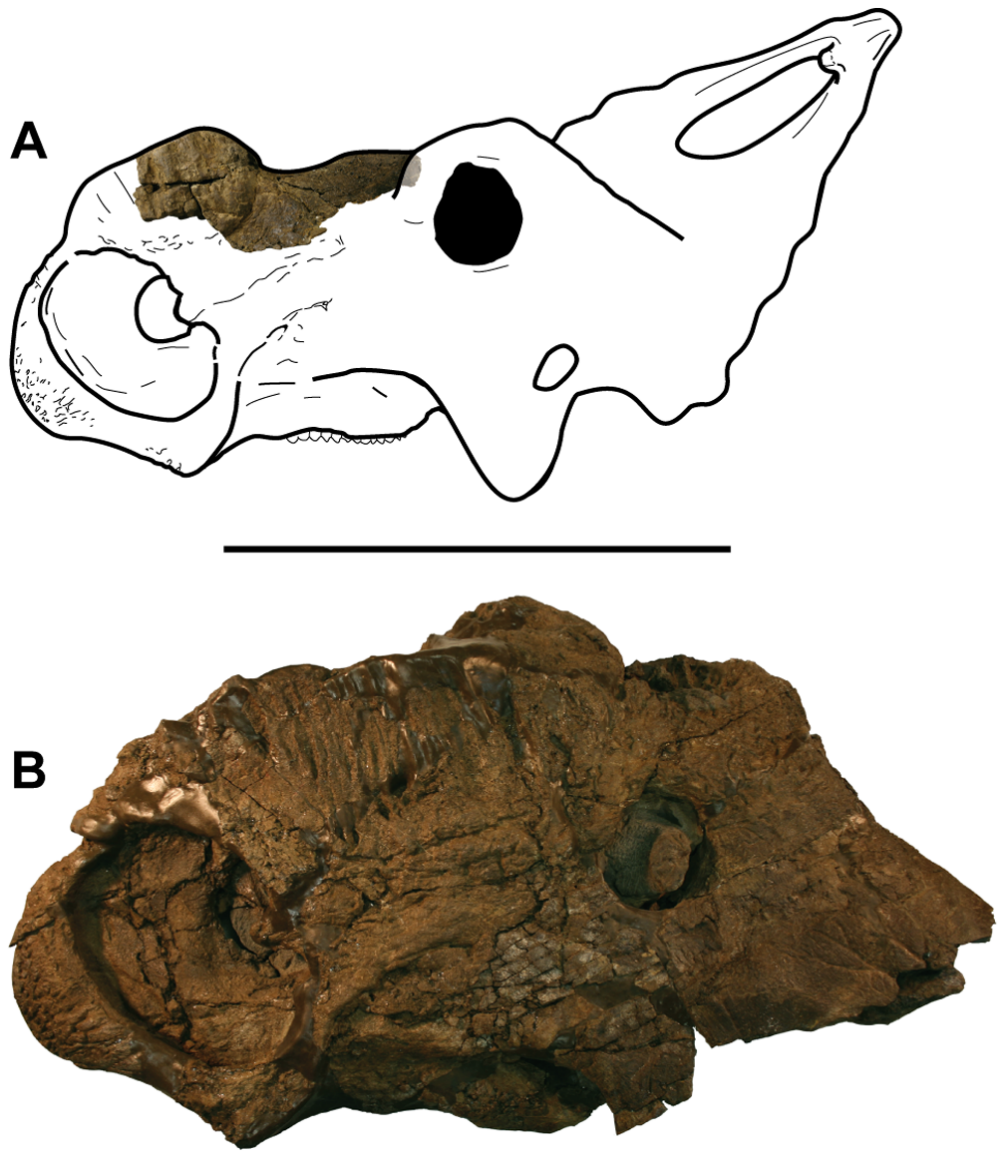
Comparison of new, immature specimen and ‘adult’ skull of *Pachyrhinosaurus perotorum*. A. DMNH 21460, *Pachyrhinosaurus perotorum* incomplete nasal superimposed on line drawing of hypothetical immature individual skull. B, DMNH 22558, *Pachyrhinosaurus perotorum* incomplete skull of mature individual. Both images in left lateral view and to scale. Scale bar equals 50 cm.

The maximum anteroposterior length of the incomplete nasal is 270 mm from the anterior broken surface through the nasal horn to the posterior margin of the bone. Maximum mediolateral width of the specimen is 115.9 mm, and maximum dorsoventral height measured from the most ventral preserved portion of the rostrum lateral surface is 127 mm. The largest immature nasal of *Pachyrhinosaurus lakustai* illustrated by Currie et al. [4] (TMP.1989.55.1342) is approximately 230 to 240 mm in length, although it is missing the narial bridge portion of the anterior end of the nasal. This suggests DMNH 21460 came from a somewhat larger individual than represented by the known immature nasals of *P. lakustai*. Comparison between DMNH 21460 and the more complete skull of a mature *P. perotorum* (DMNH 22558) suggests the new specimen came from an individual approximately two-thirds or more ‘adult’ size (Figure 4).

The nasal horn is incomplete, consisting only of the posterior part of the structure. The border of the horn is clearly defined posteroventrally and posteriorly by its bulged, raised edge, and by a narrow band of numerous small foramina and neurovascular grooves immediately adjacent to the bulged rim that parallel the raised surface (Figure 3A through F). This bordering band was termed the basal sulcus by Hieronymus et al. [10]. The preserved portion of the nasal horn measures 110 mm from its dorsal tip to the most ventral remnant of the basal sulcus on the left side, and 126.5 mm from the most anterior broken surface to the most posterior point of the basal sulcus on the midline. The nasal horn is widest ventrally (greatest breadth across the preserved parts is 76.5 mm) and narrows dorsally (Figure 3G and H). This contrasts with the nasal boss of DMNH 22558, a large, mature skull of *Pachyrhinosaurus perotorum* in which the nasal boss is widest dorsally. The cross section revealed by the anterior break through the specimen shows the bone in the lower half of the horn is dense and relatively solid, while the dorsal half is comprised of coarse spongy bone. The dorsal surface of the horn rises 55 mm above the posterior midline point of the basal sulcus, and 98 mm above the posterior edge of the nasal along the frontal contact. The rounded dorsal surface is incised by an undulating sulcus ranging from 2.5 mm to nearly 13 mm wide, and from 1.5 mm to 7.5 mm deep. The median sulcus is limited to the anterior part of the preserved horn, and the inner surfaces of the sulcus are marked by small foramina and an unfinished texture compared to the surrounding horn surface. The lateral surfaces of the horn are marked by several dorsoventrally aligned, low ridges separated from one another by shallow grooves, which together produce the palisade texture previously associated only with fully developed nasal bosses of larger, relatively mature individuals of *Pachyrhinosaurus* [1], [2], [3], [4], [5]. The presence of lateral palisade texture, combined with the bulging lateral surfaces and beginning of a median sulcus or separation on the dorsal surface suggests the nasal ornamentation of DMNH 21460 could be described as an incipient or ‘proto’ nasal boss.

The dorsal surface of the nasal slopes posteroventrally from the basal sulcus before flattening out to a nearly horizontal but transversely convex surface halfway between the nasal boss and the posterior edge of the nasal. The posterior edge of the nasal at and immediately lateral to the midline is a nearly vertical surface marked with faint pits and ridges for contact with the anterior end of the frontal and perhaps the prefrontal. The posterior rim of the nasal curves posterolaterally from the midline in a sinuous fashion to its most posterior point, then curves laterally and anterolaterally to round out the posterolateral edge of the bone (Figure 3E and F). The posterolateral edge of the nasal is dorsoventally thick (1 cm or more), smooth, and instead of having a vertical contact surface it is smoothly rounded off. The sloped surface immediately posterior to the boss is marked by numerous small (≤1 mm) foramina that intersect the surface at a steep or perpendicular angle, and short (1 cm or less), shallow grooves without an apparent preferred orientation. The more horizontal, flattened, posterior part of the nasal has a noticeably different texture. Here the bone surface is faintly and evenly rugose with a high density (20 to 28 foramina per square centimeter) of very small foramina. There are also several larger (1 to 2 cm long) vascular grooves in this area, some of which intersect to form weak branching patterns. The lateral border of this rugose patch is sharply defined by a low ridge that begins even with the midpoint between the nasal boss and the posterior edge of the nasal and curves posteromedially toward the rounded posterior margin of the nasal. The ridge tapers out as it reaches the edge of the nasal. The surface texture of the nasal lateral to the ridge is completely smooth, with no foramina or neurovascular grooves marking the surface. This is in stark contrast to all the other surfaces on the specimen.

## Results and Discussion

### Ontogenetic Implications

The numerous specimens of *Pachyrhinosaurus lakustai* from the Pipestone Creek locality of Alberta currently provide the broadest series of ontogenetic ‘snapshots’ for *Pachyrhinosaurus* [4]. The sample includes a number of isolated nasals from immature individuals of different developmental stages that formed the basis for understanding growth and modification of the nasal ornamentation from ‘typical’ immature centrosaurine nasal demihorns to the highly derived nasal boss of *Pachyrhinosaurus* [4]. There are differences in DMNH 21460 (*P*. *perotorum*) compared to the growth pattern inferred for *P. lakustai* that may provide additional information in reconstructing ontogenetic transformations in pachyrhinosaurs.

### Separation of Nasal Horn and Posterior Edge of Nasal

The nasal demihorns in immature *P. lakustai* span almost the full anteroposterior length of the nasal, leaving only a short anterior process to meet the nasal process of the premaxilla and a sloped, sculptured, triangular area between the demihorn and the posterior prefrontal-frontal contact [4]. The sculptured area reported in immature *P. lakustai* nasal demihorns may correspond to the rugose patch posterior to the nasal horn in DMNH 21460. The distance between the nasal horn and posterior rim of the nasal in DMNH 21460 is proportionally greater than in specimens of *P. lakustai* [4] (Figure 3A through D). The proportions of the posterior part of the nasal relative to the incipient nasal boss in DMNH 21460 (Figure 3I) more closely resemble those of more basal centrosaurines such as *Styracosaurus* (CMN 86–554) and some immature specimens of *Einiosaurus* [15] than fully mature *Pachyrhinosaurus*.

DMNH 21460 is the only immature nasal currently known from the Kikak-Tegoseak Quarry, and without a greater sample size we are forced into speculation when offering explanations for the relatively wide separation between the nasal boss and posterior end of the nasal in *Pachyrhinosaurus perotorum*. The nasal horn might have originated from a more anterior location than in *P. lakustai*, though this seems tenuous given the apparently conservative nature of nasal horn ontogeny in early-stage centrosaurines [4], [12], [15]. A second hypothesis is that the posterior part of the nasal underwent rapid anteroposterior elongation during development just prior to posterior-ward growth of the nasal boss.

### Nasal Boss Development

DMNH 21460 preserves a mix of features that may provide refinement to the sequence of ontogenetic stages and transformations inferred for the development of the nasal boss in *Pachyrhinosaurus* [4]. Table 1 lists five ontogenetic stages that approximate the division of descriptions and discussion of nasal boss ontogeny derived from Currie et al. [4]. DMNH 21460 exhibits some Stage 3 features such as a thickened, incipient nasal boss, and limited development of lateral ridges bounding a median depression (expressed as a median sulcus) on the dorsal surface. DMNH 21460 also has Stage 4 features including complete fusion of the right and left nasals back to their posterior margins, ventral margins of the boss defined by a basal sulcus, and lateral surfaces marked by a palisade texture of parallel dorsoventrally oriented ridges and grooves. Yet there are also features that are either not encompassed by any of the stages or reported specimens of *P. lakustai*, such as apparent lengthening of the posterior part of the nasals and separation of the dorsal zone of sculptured bone from the nasal boss, or they are not synchronous with the framework of stages and transformations reported by Currie et al. [4].

**Table 1:** Ontogenetic stages of nasal boss development in *Pachyrhinosaurus* (Stage 1 youngest), based upon descriptions and inferences of Currie et al. [4].

Growth Stage	Characteristics
Stage 1	low, paired demihorns with minimal lateral texturing in form of neurovascular grooves
	demihorn essentially covers nasal from anterior process to posterior edge
	posterior surface of demihorn not strongly deflected medially and only slightly sculptured
Stage 2	isometric growth of paired (not fused) demihorns, that extend most of the length of nasal
	lateral surface texturing with larger neurovascular grooves with variable orientation
	posterior sculptured surface larger, more distinctly sculptured; and deflected medially
Stage 3	at least two-thirds ‘adult’ size
	fusion of nasals begins anteriorly and progresses posteriorly?
	thickening of lateral surfaces, “broad tumid expansion” of Currie et al [4] of demihorns into an incipient boss
	boss precursor enlarges posteriorly but not yet to posterior edge of nasal
	posterior sculptured area apparently coalesced into expanding incipient boss
	dorsal margins with lateral ridges bounding medial depression
Stage 4	lateral surfaces of boss migrate rapidly away from midline, accompanied by rapid deposition of spongy bone between them to form obvious boss structure
	boss grows rapidly posteriorly to or nearly to posterior edge of nasals
	boss defined ventrolaterally by distinct basal sulcus
	lateral surfaces show signs of palisade texture comprised of parallel dorsoventral-oriented ridges and grooves
Stage 5	boss mediolaterally as wide or wider dorsally than ventrally along basal sulcus
	full nasal boss extends from premaxillary process to posterior edge of nasals, may contact supraorbital bosses in *P. canadensis* and *P. perotorum*
	lateral surfaces extensively marked by palisade texture of dorsventrally oriented grooves and ridges
	remodeling of boss dorsal surface

The combination of morphologies in DMNH 21460 suggests either an additional stage of development should be recognized in the ontogeny of the nasal boss of *Pachyrhinosaurus*, or that the ontogenetic pathway of nasal boss development in *P. perotorum* was notably different from that of *P. lakustai*. We are not ready to embrace the idea of large differences in the ontogenetic development of this derived structure in such closely related taxa until a larger sample of *P. perotorum* crania provides stronger evidence for it. We instead submit a revised hypothesis of nasal boss development in *Pachyrhinosaurus* that incorporates the data present in DMNH 21460 and the timing of certain morphological expressions relative to other ontogenetic transformations.

Table 2 lists a new hypothesis of ontogenetic stages for the development of the nasal boss of *Pachyrhinosaurus*, in which an additional stage is required to encompass the revised onset of morphologies seen in DMNH 21460. In this new hypothesis of nasal boss development, upon attaining at least two-thirds ‘adult’ size (Stage 3) the nasals begin to fuse to one another and the demihorns begin to transform. The nasal horn then begins to thicken laterally, undergoing the “broad tumid expansion” described by Currie et al. [4]. This is reflected in the sample of *Pachyrhinosaurus lakustai* specimens by individuals such as TMP 1989.55.256 [4]. DMNH 21460 represents an intermediate point in ontogeny (Stage 4) between specimens such as TMP 1989.55.256 and full adult crania such as represented by DMNH 22558. Stage 4 is still the point in ontogeny that sees the development of a basal sulcus around the ventral edge of the nasal boss, as well as the onset of the first palisade ridges and grooves on the lateral surfaces of the boss. The new Alaskan specimen shows that it is around this time as well that the nasals are fused along their complete length, and that there may have been a rapid lengthening of the nasal posterior to the incipient nasal boss. Only later in the next stage (Stage 5) do the lateral edges of the nasal ornamentation migrate rapidly outwards to form the true, mediolaterally wide nasal boss. It is also at this time that the nasal boss expands posteriorly, eventually overgrowing or incorporating the part of the nasal that was previously marked by the rugose, sculptured patch seen in DMNH 21460. We designate an additional stage (Stage 6) as ‘full-adult’ stage, in which the nasal boss is as wide or wider dorsally than ventrally, the nasal boss extends to or past the prefrontal and frontal contacts to nearly or actually contact the supraorbital bosses, and the lateral surfaces of the boss are extensively marked by clear palisade-texture ridges and grooves. It is possible that older individuals may also see extensive remodeling of the dorsal surface of the boss, as reported by Currie et al. [4].

**Table 2:** New hypothesis of ontogenetic stages for the nasal boss of *Pachyrhinosaurus* based on new information (marked by asterisks) derived from DMNH 21460, an incomplete nasal from an immature *Pachyrhinosaurus perotorum* individual from Alaska.

Growth Stage	Characteristics
Stage 1	low, paired demihorns with minimal lateral texturing in form of neurovascular grooves
	demihorn essentially covers nasal from anterior process to posterior edge
	posterior surface of demihorn not strongly deflected medially and only slightly sculptured
Stage 2	isometric growth of paired (not fused) demihorns, that extend most of the length of nasal
	lateral surface texturing with larger neurovascular grooves with variable orientation
	posterior sculptured surface larger, more distinctly sculptured; and deflected medially
Stage 3	at least two-thirds ‘adult’ size
	fusion of nasals begins anteriorly and progresses posteriorly?
	thickening of lateral surfaces, “broad tumid expansion” of Currie et al [4] of demihorns into an incipient boss
Stage 4	distinct basal sulcus defines ventral margins of nasal horn/incipient boss
	dorsoventrally oriented ridges and grooves begin to develop on lateral surfaces of horn/incipient boss, producing palisade texture
	*rapid anteroposterior lengthening of nasal between incipient boss and posterior edge of nasals
	*nasal fusion through horn/incipient boss and dorsal surface of nasal complete to posterior edge
	*sculptured, rugose patch on posterior end of nasal appears, separate from nasal horn/incipient boss
Stage 5	lateral surfaces of nasal boss migrate rapidly away from midline, accompanied by rapid deposition of spongy bone between them to form true boss structure
	*boss grows posteriorly to or nearly to edge of nasals, overgrowing or incorporating rugose patch on dorsal surface of nasal
Stage 6	boss mediolaterally as wide or wider dorsally than ventrally at basal sulcus
	full nasal boss extends from premaxillary process to posterior edge of nasals, may contact supraorbital bosses in *P. canadensis* and *P. perotorum*
	lateral surfaces extensively marked by palisade texture of dorsventrally oriented grooves and ridges
	remodeling of boss dorsal surface

### Integument Reconstruction

**Figure 5:**
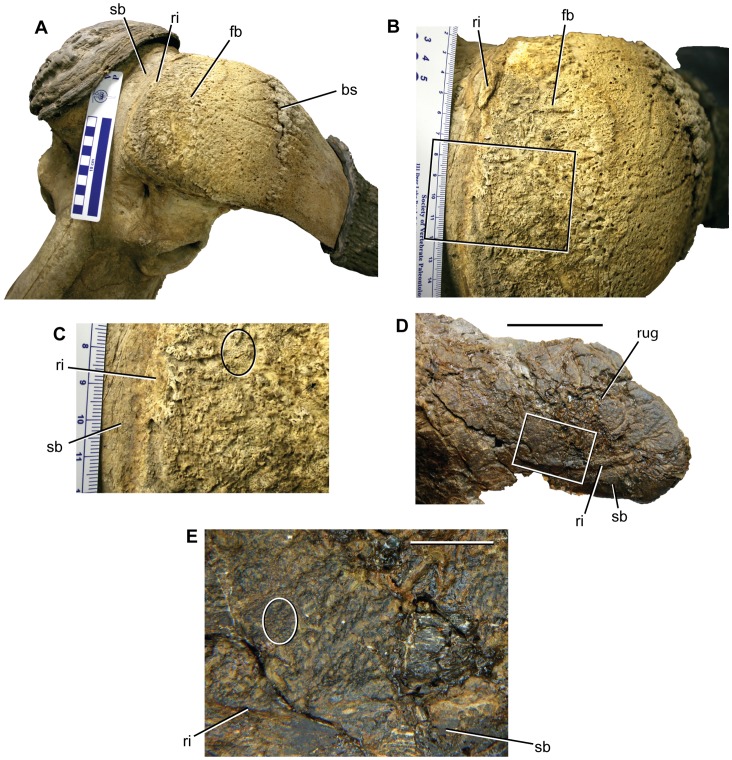
Osteological correlates for a thick cornified pad in *Synceros caffer* and *Pachyrhinosaurus perotorum*. A. DMNH 2013, *Synceros caffer* skull in left lateral oblique and dorsolateral view, showing exposed left frontal boss and proximal base of osseous horn core. B. Same specimen in closer, dorsal view centered on rugose frontal boss area. C. Close up of area inside rectangle in B. D. DMNH 21460, *Pachyrhinosaurus perotorum*, close-up of posterior end of nasal in left lateral and slightly dorsal view. E. Close up of area inside rectangle in D. Ellipses in C and E surround clusters of similar densely spaced, small foramina. Abbreviations: bs, basal sulcus; fb, frontal boss; ri, ridge separating rugose area from smooth bone; rug, rugose patch on dorsal surface of nasal; sb, smooth bone surface. Scale bar in A through C shows divisions in cm. Scale bar in D equals 5 cm. Scale bar in E equals 1 cm.

A recent study by Hieronymus et al. [10] examined craniofacial bone morphology, surface textures, and histological evidence for integument type in centrosaurine taxa as compared to extant taxa with a variety of integumentary tissues and structures. In that work, several osteological correlates were identified in the study sample that led to models of dermal tissue type, structure size, and position on several centrosaurine taxa, with particular focus on *Pachyrhinosaurus*. It was also determined that the nasal boss of *Pachyrhinosaurus lakustai* shared similarities with the frontal boss of the muskox (*Ovibos moschatus*), and to a lesser extent also with the frontal horn bosses of the African buffalo (*Synceros caffer*) (Figure 5A through C). Based on those similarities, it was determined that the dorsal surface of the nasal boss of mature *Pachyrhinosaurus* bore a thick, muskox-like, cornified pad, and that the palisade pattern of ridges and grooves on the lateral surfaces of the nasal boss were thought to have supported a thick cornified sheath growing at a shallow angle from the boss [10]. Growth direction of the sheath and cornified pad were also postulated to be parallel to the orientation of the ridges and grooves on the lateral surfaces of the boss [10].

**Figure 6:**
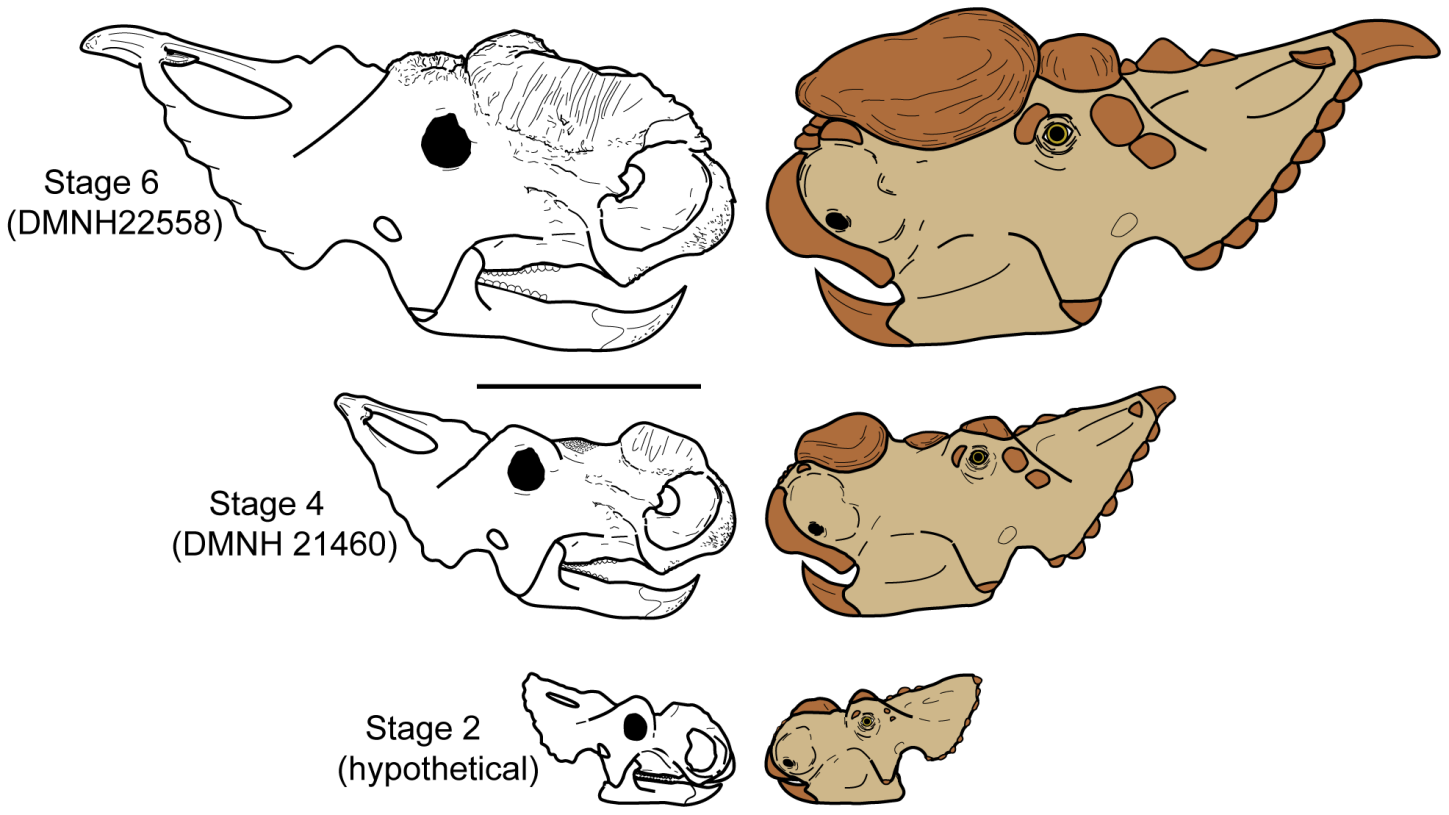
*Pachyrhinosaurus perotorum* skulls and integument reconstruction through ontogeny. Hypothesis of craniofacial changes in osteology and integument through some of the ontogenetic stages of *Pachyrhinosaurus perotorum* listed in Table 2. Stage 2 skull based on juvenile *P. lakustai* of Currie et al. [4]. Stage 4 skull based on DMNH 21460. Stage 6 skull based on DMNH 22558 [5]. Right-side ‘life’ reconstructions based on hypotheses of *Pachyrhinosaurus* integument structures of Hieronymus et al. [10], and new data from DMNH 21460. Light tan indicates ‘normal’ skin, while dark brown indicates cornified scales, horns, sheaths, or other cornified tissue. Scale bar equals 50 cm.

The presence of a distinct basal sulcus and the lateral palisade texture on the nasal horn of DMNH 21460 indicate that a thick, cornified horn sheath was present well before the formation of a dorsal cornified pad on the nasal boss. This is also supported by the orientation of some of the foramina on parts of the horn that intersect the surface at tangential angles, producing short grooves and sulci in the bone surface. If the palisade grooves and ridges on the lateral sides of the incipient nasal boss are a reliable indication of the direction of growth, given the cross-sectional shape of the nasal horn in DMNH 21460 (Figure 3G and H), in this stage of life the nasal of *P. perotorum* bore a substantial anteroposteriorly elongate but mediolaterally narrow nasal horn (Figure 6).

The dorsal rugose patch on the nasal posterior to the incipient nasal boss of DMNH 21460 is a feature that is either not present, or not recognized on specimens of *Pachryhinosaurus lakustai* [4], [10]. The rugose patch is located in the place occupied by the bulbous posterior end of the nasal boss in more mature individuals (DMNH 22558) [5]. This posterior end of a mature nasal boss is marked by anteroposteriorly oriented grooves and fins in specimens of *P. lakustai* and *P. perotorum* [4], [5]. This surface texture was interpreted to show the growth direction of a thick cornified pad on the dorsal surface of the nasal boss by Hieronymus et al. [10]. The separation of the incipient nasal boss and the sculptured rugose patch on the posterior part of the nasal in DMNH 21460 shows there was a posteriorly-located cornified skin structure on the nasal that was not originally connected to the nasal horn sheath.

The question is: what sort of cornified integumentary structure covered the rugose patch on the posterior nasal? The patch can be compared to the osteological correlates listed by Heironymus et al. [10] to determine the nature of the skin structure located on the posterior nasal. The surface of the rugose patch is faintly pitted and sculptured, bears a few neurovascular grooves with no preferred orientation, and is penetrated by numerous small foramina that appear to intersect the surface at near right-angles. The lateral margin of this textured zone is defined by a raised ridge, with very smooth bone lateral to the ridge.

We examined the frontal boss of a modern specimen of *Synceros caffer* (DMNH 2013) (Figure 5A through C) to compare the osteological textures and structure to that on DMNH 21460. The proximal part of the *Synceros* frontal boss, adjacent to the midline, has a rugose texture similar to that in DMNH 21460 (Figure 5D). The frontal boss in *Synceros* is markedly separated from a median strip of smooth bone by a raised ridge (Figure 5B and C), much as the rugose patch in DMNH 21460 is separated from a lateral area of very smooth bone (Figure 5D and E). Closer inspection of the rugose area of the *Synceros* frontal boss reveals small patches of bone penetrated by densely spaced, very small foramina (Figure 5C), a feature also seen in DMNH 21460 (Figure 5E). The similarity between the two is additional evidence for a thick cornified pad such as that on the frontal boss of *Synceros caffer* being present on the posterior end of the nasal during an intermediate stage of ontogeny in *Pachyrhinosaurus perotorum* (Figure 6). This cornified pad may have been subsequently incorporated into the nasal boss integument as the boss expanded posteriorly later in ontogeny.

### Conclusions

Material collected from the Kikak-Tegoseak Quarry of Alaska’s North Slope continues to produce new and unexpected data regarding the centrosaurine ceratopsid *Pachyrhinosaurus perotorum*. The discovery of a relatively immature nasal (DMNH 21460) reveals a more complicated craniofacial ontogeny in *Pachyrhinosaurus* than previously thought. The nasal boss of *Pachyrhinosaurus perotorum*, a structure homologous to the more erect nasal horn of more basal centrosaurine ceratopsids, underwent a point in ontogeny in which the two nasal bones were fully fused to one another and bore an anteroposteriorly long but mediolaterally narrow horn or incipient nasal boss. The long, low incipient boss bore a thick horn sheath that grew in a dorsal direction as indicated by the palisade texture on the lateral surfaces of the incipient boss. The nasal posterior to the incipient nasal boss may also have undergone rapid anteroposterior elongation prior to the onset of full nasal boss formation. Based on work that found osteological correlates of integument between centrosurine and modern taxa [10], and comparison to the frontal boss osteology of a modern *Synceros caffer* specimen (DMNH 2013), the evidence supports the hypothesis that a thick cornified pad separate from the nasal horn sheath was present on the posterior part of the nasals of immature *Pachyrhinosaurus perotorum*, prior to development of a full nasal boss. The lateral sheath originally covering the nasal horn and the posterior cornified pad may have combined only later in ontogeny to form a compound epidermal covering over the full nasal boss of mature *Pachyrhinosaurus perotorum*.
